# Does early surgery prevent Postoperative ICU admission after surgery for the fracture of the hip. Nested case control study of 911 patients

**DOI:** 10.1016/j.amsu.2020.12.017

**Published:** 2020-12-17

**Authors:** Obada Hasan, Laraib Mazhar, Umar Rabbani, Amna Rabbani, Fatima Mahmood, Shahryar Noordin

**Affiliations:** a Orthopaedic & Rehabilitation Department, University of Iowa, United states; b Department of Medicine, The Aga Khan University hospital, Pakistan; c The Aga Khan University, Pakistan; d Department of surgery, Section of orthopedics, The Aga Khan University hospital, Pakistan

**Keywords:** Hip fracture, Surgery, Early, Late, Postoperative ICU, Morbidity, Nested case-control, Complication

## Abstract

**Introduction:**

Since most hip fractures are treated surgically, it is imperative to find an optimum fracture-to-surgery time to decrease the potential complications and enhance postoperative outcomes. In comparison to the vast plethora of literature available on surgical delay and its implications on mortality, very little, if any, research is available on the impact of delayed surgery on postoperative ICU admission. The primary objective of our study is to examine the factors influencing post-surgical ICU admission in order to work on preventive strategies to reduce the potential associated morbidity.

**Material and methods:**

Investigators did a nested case control study in a university hospital. A case was defined as a patient who had postoperative ICU admission while controls were patients who did not have postoperative ICU admission after hip fracture surgery. The primary outcome variable was postoperative ICU admission. The exposure variable was defined as the time to surgery which was categorized into two categories; early and late; the early surgery included patients who were operated within ≤ 48 h and the late included patients who had their surgery >48 h. Information on potential confounders including age, type of the procedure and comorbidities were also obtained. Result reported in-line with STROCSS criteria.

**Results:**

A total cohort of 1084 hip fracture surgeries were performed from January 2010 to December 2018. After screening for eligibility criteria, 911 patients were eligible for the final simple logistic regression analysis (48 cases and 863 controls). Our exposure variable i.e. time from admission to surgery showed no difference between cases and controls. The odds of being treated with Hemiarthroplasty among cases admitted in ICU was 2.42 times as compared to controls (aOR = 2.42; 95% C.I. 1.21–4.86).

**Conclusion:**

Our study did not find an association between surgical delay and post-operative ICU admission after accounting for other covariates and potential confounders.

## Highlights

• There is a controversy of the benefits of early hip fracture surgery.
• Delay in surgery is postulated to carry higher risk of postoperative ICU admission.
• However, this higher risk was statistically insignificant in the current study.

## 1 Introduction

Hip fracture occurs frequently in the elderly population and is an important cause of decline in the functional status. The number of hip fractures has been on an increase as populations continue to age, and as per extrapolation from epidemiological studies more than 6 million cases per annum, world-wide, are predicted by the year 2050 [1,2]. The sheer number, along with high morbidity and mortality rates, puts an immense social and economic burden, especially in developing countries [1]. Mortality rate due to hip fractures is as high as 30% [3]. Adults aged 50 years and older have a 5- to 8-fold increased risk for all-cause mortality during the first 3 months after hip fracture, although the increased risk can persist for up to 10 years [4].

Since most hip fractures are treated surgically, it is imperative to find an optimum fracture-to-surgery time to decrease the potential complications and enhance postoperative outcomes. Literature on the correlation between surgical delay and postoperative complications is inconclusive with some claiming a beneficial effect of early surgery on patient mortality [5], whereas others not showing a statistically significant correlation between the two [[6], [7], [8], [9]]. Current guidelines, however, recommend early surgery, if possible, because several studies have demonstrated improved outcomes, with no documented adverse effect of operating within 48 h, especially in otherwise physiologically healthy patients [[10], [11], [12], [13], [14]]. On the contrary, several deleterious effects have been reported with delayed surgery including, but not limited to, prolonged length of stay [[15], [16], [17]], pressure ulcers [6,10], arrhythmias [16], poor postoperative hip function [18] and increased mortality [12,19]. However, it is equally important to note that impetuous surgery without proper pre-operative stabilization can also lead to adverse outcomes as most of these patients are elderly with multiple comorbidities [9].

In comparison to the vast plethora of literature available on surgical delay and its implications on mortality, very little, if any, research is available on the impact of delayed surgery on postoperative ICU admission. The primary objective of our study is to examine the factors influencing post-surgical ICU admission in order to work on preventive strategies to reduce the potential associated morbidity.

## 2 Methodology

### 2.1 Study design and study setting

A hospital-based nested case control study was conducted at the Musculoskeletal and Sports Medicine Service Line at the Department of Surgery-a tertiary care referral private university hospital which is a Joint Commission International (JCI) accredited. The study was conducted after institutional Ethical Review Committee clearance was obtained and was registered at clinicaltrials.gov with UIN. Medical records were reviewed for admitted patients from January 2010 to December 2018. The research team comprised of specialists in the fields of orthopedic surgery, epidemiology and biostatistics. Data collectors were interns, who were graduates of the same institute and trained in data collection process and management.

### 2.2 Study population and eligibility criteria

Investigators identified cases from a retrospective cohort of patients who had undergone hip fracture surgery. Cases were those patients who were operated for hip fracture and admitted to the ICU postoperatively. The controls were selected from same population which gave rise to the cases, and sampling of controls was independent of the exposure of interest in order to minimize selection bias and increase the internal validity of the study. Two data collectors were blinded from the objectives of the study to further minimize any sort of information/misclassification bias. The data was collected and reported in line with STROCSS criteria [36]. Patients older than 50 years of age and both genders who had hip fracture procedures were included. Furthermore, any patient with missing data in either the primary exposure or the outcome was excluded.

### 2.3 The primary outcome, exposure, covariates and potential confounders

As stated earlier, for the purpose of this study, a case was defined as a patient who had postoperative ICU admission while controls were patients who did not have postoperative ICU admission after their hip fracture surgery. The primary outcome variable was postoperative ICU admission. The primary exposure was the time to surgery which was categorized into two categories; early and late; the early surgery included the individuals who were operated within ≤ 48 h and the late included patients who had their surgery after 48 h of their admission. The cut point of 48 h was based on recent systematic review and a metanalysis [37]. The covariates included gender, procedure, mechanism of injury, type of fracture, type of anesthesia, CCI status and ambulation status at discharge. Information on potential confounders including age, type of the procedure and comorbidities was also obtained.

### 2.4 Statistical analysis

Data was analyzed using STATA version 14. The demographics and background characteristics were assessed between the cases and controls. Qualitative variables were reported as frequency and each assessed for comparability between cases and controls by Chi-square and simple logistic regression.

Univariate analysis using simple logistic regression was done reporting crude odds ratio (OR), confidence interval (C.I.) and *p value*. After a univariate analysis, we included the primary exposure and all variables with *p* value of 0.25 or less for the multivariable model where we followed a stepwise approach reporting adjusted OR, C.I. with *p* value 0.05 or less considered as significant. Plausible associations were checked in the final model between age and the primary exposure, age and ambulation status as well as between age and procedure. We did exact matching between cases and controls of the procedure (DHS, Hemiarthroplasty, THR and others) with 1:5 ratio yielding 48 cases and 240 controls.

## 3 Results

### 3.1 Description of study participants

**Fig. 1:**
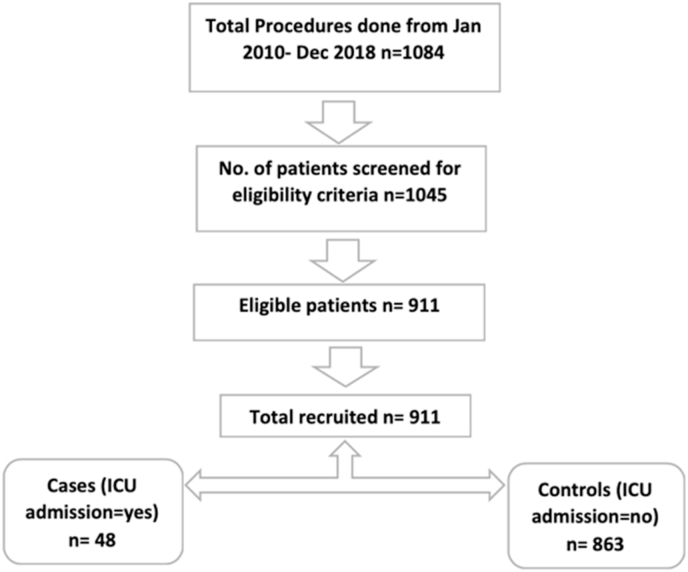
Flow chart of patient's participations

A total number of 1084 of hip fracture surgeries were performed from January 2010 to December 2018. After screening for eligibility criteria, 911 patients were eligible for the final simple logistic regression analysis (48 cases and 863 controls). Flowchart of data extraction is shown in Fig. 1. Our exposure variable i.e. time from admission to surgery showed no difference between cases and controls with *p* value of 0.31 (Table 1).

**Table 1:** Demographic and clinical characteristics of cases and controls.

Variables	Cases n = 48	Controls n = 863	*p* value*	Variables	Cases n = 48	Controls n = 863	*p* value
	N (%)	N (%)			N (%)	N (%)	
**Age (Years)**			0.79	**Type of Fracture**			0.07
*50–65*	13(27%)	228(26%)		*IT*	18(38%)	461(53%)	
*65–80*	25(52%)	481(56%)		*NOF*	28(58%)	359(42%)	
*80+*	10(21%)	154(18%)		*Sub Troch*	2(4%)	43(5%)	
**Sex**			0.07	**Type of Anesthesia**			0.13
*Male*	25(52%)	327(38%)		*GA*	13(18%)	134(22%)	
*Female*	23(48%)	536(62%)		*Regional*	58(82%)	465(78%)	
**Procedure**			0.06	**Type of Procedure**			0.46
*DHS*	16(33%)	458(53%)		*Elective*	24(50%)	480(56%)	
*Hemiarthroplasty*	18(38%)	208(24%)		*Emergency*	24(50%)	383(44%)	
				**CCI**	0.08		
*THR*	8(17%)	106(12%)		*Mild*	2(4%)	61(7%)	
*Others (PFP/Cannulate Screws/IMN)*	6(12%)	91(11%)		*Moderate*	9(19%)	279(32%)	
				*Severe*	37(77%)	523(61%)	
**Time from ER to Surgery**			0.31	**Ambulation status at Discharge**			0.92
*Early* < = *48 h*	16(5%)	351(95%)		*FWB*	20(6%)	353(94%)	
*Late* > *48 h*	32(6%)	512(94%)		*NWB*	28(5%)	510(95%)	
**Mechanism of Injury**			0.43	**Mortality**			<0.01
*Ground level fall*	42(88%)	786(91%)		*Dead*	11(23%)	5(1%)	
*Others (higher energy)*	6(12%)	77(9%)		*Alive*	37(77%)	858(99%)	

*Proportions in the two groups are compared using Wald χ2 test from simple logistic regression model, *p* value of ≤0.05 is significant.

Abbreviations: DHS: Dynamic Hip Screw, THR: Total Hip Replacement, PFP: Proximal Femur Plate, IMN: IntraMedullary Nail, GA: General Anesthesia, IT: InterTrochanteric, NOF: Neck of Femur, CCI: Charlson Comorbidity Index, FWB: Full Weight Bearing, NWB: Non-Weight Bearing.

### 3.2 Univariate analysis

We observed that gender (*p* value 0.052), procedure (*p* value 0.075), type of fracture (*p* value 0.07), type of anesthesia (*p* value 0.17), CCI status (*p* value 0.061) were found to be significant at univariate level (Table 2). The odds of being a female amongst cases was 44% less as compared to controls (OR = 0.56; 95% C.I. 0.31–1.01). Furthermore, odds of receiving regional anesthesia were 55% higher among cases as compared to controls (OR = 0.45; 95% C.I. 0.13–1.50). The odds of severe CCI status was significantly higher among cases as compared to controls (OR = 2.15; 95% C.I. 0.51–9.18).

**Table 2:** Unconditional logistic regression analysis at the Univariate level for the factors associated with ICU admission reporting crude odds ratio OR and 95% C.I.

Variables	OR(96% C.I.)	*p* value (0.25)
*Age(years)*	0.99(0.96–1.03)	0.72
**Time from ER to Surgery**		0.31
*Early* < = *48 h (Ref.)*	1	
*Late* > *48 h*	1.37(0.74–2.53)	
**Sex**		**0.05**
*Male (Ref.)*	1	
*Female*	0.56(0.31–1.01)	
**Procedure**		**0.08**
*DHS(Ref.)*	1	
*Hemiarthroplasty*	2.47(1.24–4.96)	
*TH*	2.16(0.90–5.18)	
*Others (PFP/Can Screws/IMN)*	1.89(0.72–4.95)	
**Mechanism of Injury**		0.42
*Ground level fall (Ref.)*	1	
*Others (higher energy)*	1.45(0.60–3.54)	
**Type of Fracture**		**0.07**
*IT(Ref.)*	1	
*NOF*	1.99(1.09–3.69)	
*Sub Torch*	1.19(0.27–5.31)	
**Type of Anesthesia**		**0.17**
*GA(Ref.)*	1	
*Regional*	0.45(0.13–1.50)	
**Type of Procedure**		0.45
*Elective (Ref.)*	1	
*Emergency*	1.25(0.70–2.241)	
**CCI**		**0.06**
*Mild (Ref.)*	1	
*Moderate*	0.98(0.21–4.67)	
*Severe*	2.15(0.51–9.18)	
**Ambulation status at Discharge**		0.92
*FWB(Ref.)*	1	
*NWB*	0.97(0.54–1.75)	

Abbreviations: Ref: Reference category, DHS: Dynamic Hip Screw, THR: Total Hip Replacement, PFP: Proximal Femur Plate, IMN: IntraMedullary Nail, GA: General Anesthesia, IT: InterTrochanteric, NOF: Neck of Femur, CCI: Charlson Comorbidity Index, FWB: Full Weight Bearing, NWB: Non-Weight Bearing.

### 3.3 Multivariable analysis

In multivariable analysis, step wise approach was conducted including the primary exposure i.e. time from admission to surgery after checking for multi collinearity. All factors, other than the procedure, were found to be highly insignificant predictors for ICU admission after controlling for other variables in the model except for the surgery. Individuals operated treated with hemiarthroplasty were more likely to have ICU admissions (Table 3). The odds of being treated with Hemiarthroplasty among cases admitted in ICU was 2.42 times as compared to controls (aOR = 2.42; 95% C.I. 1.21–4.86). All possible plausible interactions were checked and found insignificant (*p* value > 0.1). After exact matching on procedure, none of the variables, including the primary exposure of early Vs late surgery, studied was significant (Table 4).

**Table 3:** Final model after multivariable analysis for factors associated with ICU admission post hip fracture surgery.

Variables	aOR(95% C.I.)	*P*-VALUE
**Time from ER to Surgery**		0.37
*Early* < = *48 h (Ref.)*	1	
*Late* > *48 h*	1.24(0.64–2.42)	
**Procedure**		**0.01**
*DHS(Ref.)*	1	
*Hemiarthroplasty*	2.42(1.21–4.86)	
*THR*	2.18(0.91–5.24)	
*Others (PFP/Canulated Screws/IMN)*	1.87(0.72–4.93)	

aOR: Adjusted Odds Ratio. C.I.: 95% Confidence Interval. *p* value of ≤0.05 is significant.

**Abbreviations:** Ref: Reference category, DHS: Dynamic Hip Screw, THR: Total Hip Replacement, PFP: Proximal Femur Plate, IMN: IntraMedullary Nail.

**Table 4:** Conditional logistic regression analysis at the Univariate level after matching the cases and controls on procedure.

Variables	mOR(C.I)	*p* value (0.25) *
*Age(years)*	1.01(0.97–1.04)	0.79
**Time from ER to Surgery**		**0.13**
*Early* < = *48 h (Ref.)*	1	
*Late* > *48 h*	1.64(0.851–3.144)	
**Sex**		**0.06**
*Male (Ref.)*	1	
*Female*	0.55(0.300–1.031)	
**Mechanism of Injury**		0.51
*Ground level fall (Ref.)*	1	
*Others (higher energy)*	1.37(0.550–3.454)	
**Type of Fracture**		0.87
*IT(Ref.)*	1	
*NOF*	0.76(0.155–3.732)	
*Sub Torch*	0.61(0.094–4.026)	
**Type of Anesthesia**		0.91
*GA(Ref.)*	1	
*Regional*	0.96(0.483–1.910)	
**Type of Procedure**		0.41
*Elective (Ref.)*	1	
*Emergency*	1.32(0.686–2.538)	
**CCI**		**0.13**
*Mild (Ref.)*	1	
*Moderate*	1.05(0.207–5.395)	
*Severe*	2.10(0.461–9.576)	
**Ambulation status at Discharge**		0.75
*FWB(Ref.)*	1	
*NWB*	0.903(0.484–1.684)	

Abbreviations: mOR: Matched odds ratio, Ref: Reference category, GA: General Anesthesia, IT: InterTrochanteric, NOF: Neck of Femur, CCI: Charlson Comorbidity Index, FWB: Full Weight Bearing, NWB: Non-Weight Bearing.

*p value of 0.25 was selected to include as many variables as possible to the multivariable model. However, none of the variables was significant after adjusting for other covariates in the model.

## 4 Discussion

Our study did not show a statistically significant impact of delayed surgery on postoperative ICU admission (*p* value = 0.31) after accounting for other covariates and potential confounders. Plausibly, this could be due to better preoperative optimization resulting in enhanced postoperative outcomes. Despite not being the primary reason for delay, preoperative investigations and stabilization of elderly patients contributes to some of the lengthiest delays in surgery in elderly patients [22,23]. Some authors recommend delays of up to 72 h to improve outcomes in patients with multiple comorbidities [24] because early surgery prior to preoperative stabilization, in these patients, has been reported to adversely affect the outcomes and increase postoperative morbidity [14]. However, it is important to note that patients who had their surgery delayed due to medical reasons had 2.5 times increased risk of 30-day mortality compared to patients who were stable for surgery [25].

Bulk of the research in previous years has focused on surgical delay and its effects on mortality and postoperative complications in general, with very little literature on its correlation with postoperative ICU admission. Although some studies have shown an increase in hospital stay in patients who had a surgical delay of more than 48 h [16], it's unclear as to whether it included ICU stay. ICU admissions are associated with an increased financial burden [20] and worse outcomes with 1-year reported mortality of up to 76% [21].

On univariate analysis, we found that gender had a significant impact on post-op ICU stay as females were less likely to be admitted into ICU despite accounting for a greater number of hip fractures. This is one of the widely reported associations in literature which state that men have higher rates of mortality and morbidity [[26], [27], [28], [29], [30]]. General anesthesia also significantly impacted postoperative ICU admission although this association was not observed on multivariate analysis. This is in concordance with other studies which report a higher incidence of post-operative complications in patients who underwent general anesthesia as compared to spinal anesthesia [31,32]. Therefore, we suggest that spinal anesthesia be used, wherever possible, to decrease the potential risk of ICU admission following hip fracture surgery. Our study didn't show Preoperative Charlson Comorbidity Index (CCI) score to be associated with increased risk of postoperative ICU admission. This finding is in contrast to Flikweert et al. who reported that CCI≥3 was associated with increased complications [32], although the mortality rate was not higher in patients with a complication. Other studies have also reported a correlation between high CCI and mortality [21]. Sofu et al. reported post-operative ICU admission as a main determinant of hospital readmission and increased mortality [33]. Higher American Society of Anesthesiology score have also been reported with statistically significantly increased mortality [26,29,34].

At multivariable modeling, only the procedure was significantly associated with ICU admission. Our study showed patients with hemiarthroplasty had an increased risk of postoperative ICU admission. One possible explanation is that this procedure takes substantially lesser time than THR or fixation procedures making it the procedure of choice in elderly frail patients with more comorbidities and higher risk factors. The mortality rate was 23% in patients who were admitted in ICU post-surgery as opposed to 1% in controls, which was statistically significant (*p* value < 0.01). Gibson et al. reported acute hospital mortality of 33% in patients who had critical care admission with one-year mortality of 54% [35]. Outcomes depended on time between surgery and critical care admission as well as the reason for admission due to sepsis having the worst outcomes. Eschbach et al. also reported an in-hospital mortality of 26% in patients who required ICU admission for more than three days [21].

### 4.1 Strengths

As the clinical outcomes of postoperative ICU admission have been sporadically researched for obvious reasons, the nested case control design was the best study design we could rely on with such a rare outcome. Furthermore, to the authors’ knowledge, this is the first reported study, that we are aware of, which compares the effects of delayed surgery on postoperative ICU admission as a primary study question accounting for multiple confounders.

### 4.2 Limitations

Retrospective design is the main caveat of this study. Because of this, we could not consider other factors which could potentially influence ICU admissions, as well as the reason for ICU stay and type of management done. It was beyond the scope of this study to analyze the exact complications leading to ICU admission and the time spent in ICU. The reason for delay in surgery was also not documented and is another possible confounder especially if the delay was because of optimizing the patient preoperatively to reduce intra and post-operative complications. Moreover, we couldn't assess the time between injury/fracture and presentation in the hospital. Lastly, our study had a decent sample size, the number of cases was relatively small.

## 5 Conclusion

Although our study did not find an association between surgical delay and post-operative ICU admission after accounting for other covariates and potential confounders, we believe that this topic needs to be studied further to identify the predicting factors of being admitted to the ICU. This will guide the patient-physician counselling into the option of treatment.

## Ethical Approval

Yes given by the Aga Khan University Ethical Review Committee , ERC 4543 21-dec-16.

## Sources of funding

None.

## Author contribution

**Obada Hasan**: Design of the protocol, conducting the study, analysis and manuscript writing and final approval.

**Laraib Mazhar**: Manuscript editing, analysis and final approval.

**Umar Rabbani**: Data collection, Manuscript editing, analysis and final approval.

**Amna Rabbani**: Data collection, Manuscript editing, analysis and final approval.

**Fatima Mahmood**: Data collection, Manuscript editing, analysis and final approval.

**Shahryar Noordin**: Design and manuscript review and final approval.

## Research registration number

1. Name of the registry:Retrospective chart review registered at clinicaltrials. gov.

2. Unique Identifying number or registration ID:NCT04187261.

3. Hyperlink to the registration (must be publicly accessible):https://clinicaltrials.gov/ct2/show/NCT04187261.

## Guarantor

All Authors take responsibility.

## Disclaimer

None

## Financial support and sponsorship

Nil.

## Provenance and peer review

Not commissioned, externally peer-reviewed.

## Declaration of competing interest

There are no conflicts of interest.
